# Harnessing early life immunity to develop a pediatric HIV vaccine that can protect through adolescence

**DOI:** 10.1371/journal.ppat.1008983

**Published:** 2020-11-12

**Authors:** Ria Goswami, Stella J. Berendam, Shuk Hang Li, Ashley N. Nelson, Kristina De Paris, Koen K. A. Van Rompay, Sallie R. Permar, Genevieve G. Fouda

**Affiliations:** 1 Duke Human Vaccine Institute, Duke University Medical Center, Durham, North Carolina, United States of America; 2 Department of Microbiology and Immunology, School of Medicine, University of North Carolina at Chapel Hill, Chapel Hill, North Carolina, United States of America; 3 Children’s Research Institute, School of Medicine, University of North Carolina at Chapel Hill, Chapel Hill, North Carolina, United States of America; 4 California National Primate Research Center, University of California, Davis, California, United States of America; 5 Department of Pediatrics, Duke University Medical Center, Durham, North Carolina, United States of America; McGill University, CANADA

## Need for an earlylife HIV vaccine

The successful implementation of antiretroviral therapy (ART) in women living with HIV (WLWH), either for their own health or for prevention of mother-to-child transmission (MTCT), has reduced MTCT risk of HIV to <5% [1]. Yet, in 2018, worldwide, approximately 160,000 infants were infected with HIV [2]. The currently available ART-based measures to prevent MTCT in WLWH are limited by implementation challenges, such as suboptimal ART coverage of pregnant and breastfeeding women [3], poor adherence to ART [4,5] resulting in incomplete viral suppression and increased risk of drug resistance, and late presentation for prenatal care [6]. In addition, ART-based prophylactic strategies do not address the scenario of acute maternal infections that occur late during pregnancy or during the breastfeeding period [7]. While breastfeeding is critical to reduce infant mortality in resource-limited settings by providing nutrition and protection against common childhood diseases, it also contributes to >50% of new infant HIV infections. Therefore, to prevent MTCT and achieve an HIV-free generation, novel immune-based intervention strategies beyond ART need to be explored. While these immune-based strategies could be administered either to pregnant women or to infants in the form of active immunization or passive immunization, this review primarily focuses on active immunization of neonates with an HIV vaccine that can protect during early life, from breast milk transmission, and during adolescence, from sexual transmission.

**Fig 1:**
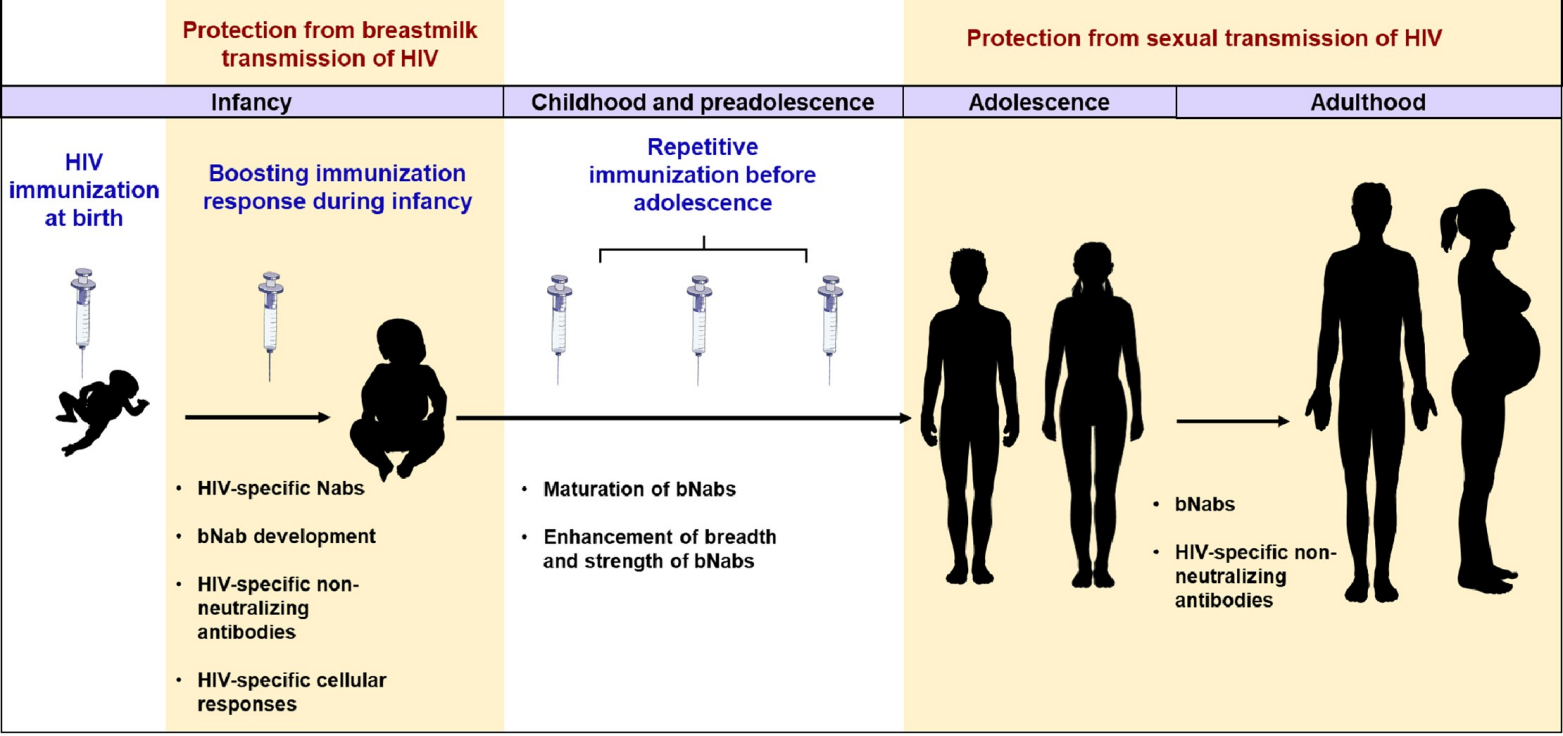
Early life vaccination to achieve protection from bimodal HIV acquisition. An HIV vaccine administered at birth with successive boosting during infancy will induce anti-HIV neutralizing and non-neutralizing antibody responses and HIV-specific cellular immunity that will reduce the risk of HIV infection via breastfeeding. Moreover, immunization started at birth and boosted during infancy, childhood and preadolescence will provide those neutralizing antibodies sufficient time to mature, undergo extensive affinity maturation, and SHM and enhance their breadth and strength prior to sexual debut. These developed bNabs will confer protection against sexual transmission of HIV during adulthood. bNabs, broadly neutralizing antibodies; SHM, somatic hypermutation.

In addition to the risk of HIV infections that occur early in life via breastfeeding, sexual transmission during adolescence and adulthood also represents a significant and ongoing mode of infection [8]. A pediatric HIV vaccine administered at birth and boosted during infancy may protect infants during the period of repetitive HIV exposure via breastfeeding. Additionally, sequential boosting through childhood and preadolescence may allow for the maturation of their immune responses and the development of broadly protective immunity prior to sexual debut, including HIV-specific broadly neutralizing antibodies (bNabs), which can neutralize a diverse variety of HIV strains by targeting conserved viral epitopes. Passive immunization with bNabs has been shown to be associated with modest and transient suppression of viremia in humans [9] and in animal models [10,11]. Consequently, elicitation of bNabs is a major goal for an efficacious HIV vaccine. Since bNab development requires years of affinity maturation and somatic hypermutation (SHM), the period between breastfeeding and sexual debut represents a unique “window of opportunity” to boost anti-HIV antibody responses at a time when the risk of HIV infection is low. Thus, a vaccine strategy initiated at birth and pursued through adolescence may protect an individual from infancy through adulthood (Fig 1).

Several recent studies have indicated that the early life immune system may present some advantages for elicitation of HIV-specific antibody responses. The purpose of this review is to summarize these studies and highlight the unique ability of the early life developing immune system to mount robust and durable immune responses against HIV, compared to adults. Additionally, the potential of harnessing neonatal immune ontogeny to develop an effective earlylife HIV vaccine is emphasized.

## Infants can develop robust and durable responses to HIV vaccination

Owing to maturational differences in the early life and adult immune systems, the ability of infants to generate vaccine-specific immune responses has traditionally been considered as impaired [reviewed in [12]]. Additionally, there are concerns around vaccine safety in infants, possible interference of passively acquired maternal antibodies with the development of protective immune responses [13], and interference of novel vaccines with commonly administered childhood vaccines [14]. These factors are further complicated by the inability of the child to provide approval of participation in the trial, challenges of obtaining parental consent for the child to participate due to perceived risks of the trial [15], and the constraint of limited blood volumes obtained from infants for immunogenicity evaluations. As a consequence, only a restricted number of infant vaccine trials have been conducted, to date. Nevertheless, increasing evidence indicates that infants can mount robust and durable immune responses following vaccination, demonstrating that earlylife immune system is not unresponsive. In fact, it is increasingly recognized that qualitative and quantitative differences between infant and adult immune system are critical for the adaptation of early-life immune system to the ex utero environment [reviewed in [16]].

Neonatal nonhuman primate (NHP) studies have provided encouraging results regarding the ability of infants to mount durable immune responses against HIV or simian immunodeficiency virus (SIV) vaccines [17–19]. In fact, the concept of vaccination at birth to protect through infancy and adolescence was highlighted by a study demonstrating that immunization of neonatal rhesus macaques (RM) with poxvirus-based SIV vaccines early after birth provided partial protection against multiple low-dose SIV challenges not only during infancy but also during adolescence [20]. Other studies have also demonstrated that HIV vaccination can induce robust antibody responses in infant RMs, although the protective roles of these antibodies were not investigated in that cohort [21].

To date, only a few pediatric HIV vaccine trials have been completed (Table 1). While none of these vaccine trials was designed to evaluate vaccine efficacy, these trials have consistently confirmed a good safety profile in infants. All these trials have indicated that vaccinated infants could develop robust and durable antigen-specific humoral and cell-mediated immune responses, despite the use of different antigens, adjuvants, and viral vectors. To determine whether infants were capable of eliciting potentially protective responses, antibody responses from infant HIV vaccinees (Pediatric AIDS Clinical Trials Group [PACTG] 230 and PACTG 326 trials) (Table 1) were compared to those of the vaccine recipients from the RV144 trial, the only adult HIV vaccine trial that resulted in moderate efficacy [22]. Immune correlate analysis of RV144 indicated an association between a higher magnitude of immunoglobulin G (IgG) responses against the envelope variable loops 1 and 2 (V1V2) with reduced risk of HIV acquisition, whereas envelope-specific immunoglobulin A (IgA) responses were associated with increased risk of HIV acquisition [23]. Infants from both trials mounted robust V1V2-specific IgG responses, yet vaccine-elicited Env-specific IgA responses were rarely detected. Interestingly, the frequency of V1V2 IgG response of infants immunized with MF-59-adjuvanted rgp120 vaccine (Chiron, United States of America) (PACTG 230 trial) was also higher than that of RV144 vaccinees, and high magnitude antibody responses were still detected more than 6 months after immunization [24]. Since RV144 vaccinees received a different vaccine regimen than PACTG vaccinees, the ability of infants to mount V1V2 IgG responses at a higher magnitude than adults was further confirmed by comparing the antibody responses between infants and adults immunized with similar vaccine regimens [25]. Additionally, the presence of maternal antibodies did not interfere with the infants’ ability to mount antibody responses [24,26], and immunization with HIV vaccine during infancy did not inhibit the ability of other childhood vaccines to induce protective antibody levels [27,28].

**Table 1:** Pediatric HIV vaccine clinical trials.

Trial	Phase	Vaccine candidates	Adjuvants	Study population	Study population age	Year of enrollment/references
PACTG 218	Phase I	rgp120-SF2 (Chiron, USA)	MF-59	Asymptomatic HIV-infected infants and children in the USA	1 month to 18 years	1993–1994 [29]
		rgp160 IIIB (MicroGeneSys, USA)	Alum			
		rgp120-MN (Genentech, USA)	Alum			
PACTG 230	Phase I	rgp120-MN (VaxGen, USA)	Alum	HIV-exposed infants born to WLWH in the USA	Birth (<72-hour age)	1993–1996 [30,31]
		rgp120-SF2 (Chiron)	MF-59			
PACTG 326	Phase I/II	ALVAC-HIV vCP205 (Sanofi-Pasteur, France)	-	HIV-exposed infants born to WLWH in the USA	Birth (≤72-hour age)	1998–1999 [32]
		ALVAC-HIV vCP1452 (Sanofi-Pasteur) AIDSVAX B/B-rgp120-MN and rgp120-GNE8 (VaxGen)	Alum			2000–2002 [26]
HPTN 027	Phase I	ALVAC-HIV vCP1521(subtype E/B) (Sanofi-Pasteur)	-	HIV-exposed infants born to WLWH in Uganda	Birth (≤72-hour age)	2006–2007 [33,34]
PedVacc 001	Phase I	MVA.HIVA (Impfstoffwerk DessauTornau, Germany)	-	HIV–negative infants born to HIV-uninfected women in Gambia	20 weeks	2010 [28]
PedVacc 002	Phase I/II	MVA.HIVA	-	HIV-exposed infants born to WLWH in Kenya	20 weeks	2010 [35]

HPTN, HIV Prevention Trials Network; PACTG, Pediatric AIDS Clinical Trials Group; PedVacc, Pediatric Vaccine; WLWH, women living with HIV.

## Development of protective broadly neutralizing antibody responses and non-neutralizing responses in young children

Although the induction of cross-clade bNab is 1 of the major goals of any HIV vaccination strategy [36,37], so far, no HIV vaccine regimen has successfully elicited such responses. Therefore, understanding the immune mechanisms behind generation of such bNab responses remains the number 1 priority in the HIV vaccination research. During natural infection, bNabs develop only in 10% to 30% of the HIV-infected adults after several years of infection and are associated with extensive SHM and affinity maturation [38,39]. In contrast, HIV-infected children can develop broad neutralization earlier than adults [40–43] and exhibit increase in neutralization breadth and potency over time [41,44]. Moreover, compared to adults, where plasma neutralization breadth is driven by antibodies of limited specificities, neutralization breadth in children is often achieved via polyclonal epitope specificity [40,45]. To date, the 2 bNabs isolated from children demonstrated absence of extensive SHM [43,46]. The mutational changes that are critical for bNab functionality were revealed to be distinct in pediatric population compared to adults [47]. Mutations important for the functional activity of 1 isolated pediatric bNab were primarily found in the heavy chain complementarity-determining region 2 (HCDR2) and light chain complementarity-determining region 1(LCDR1), in contrast to adult bNabs, where major determinant of breadth reside in the heavy chain complementarity-determining region 3 (HCDR3) [47]. For the other isolated pediatric bNab, unlike adult bNabs, indels in the heavy chain framework 3 region (HFR3) seemed to be critical for neutralization breadth [43]. Additionally, neutralization breadth in children was found to be associated with several immune parameters such as T follicular helper (Tfh) cells, circulating T follicular regulatory (Tfr) cells [48], and T helper cell 2 (Th2) cytokine interleukin 5 (IL-5) levels in plasma [49]. These findings suggest that neutralization breadth in children is driven by distinct mechanisms than in adults. Therefore, to augment our understanding of the advantage of early immune landscape on the development of neutralization breadth and to provide possible leverage in the quest of protective pediatric HIV vaccination regimens, further isolation, and characterization of pediatric bNabs will be crucial. Understanding the immune mechanisms, evolutionary pathways, and mutational changes that are critical for bNab development and activity in early life will be crucial for designing effective HIV vaccines.

While elicitation of bNabs remains a priority of HIV vaccine development, a combination of neutralizing and non-neutralizing effector responses might be crucial for an efficacious vaccine. A recent study has indicated that polyfunctional antibody responses are predictive of bNab development [50], suggesting that the pathways for induction of neutralizing and non-neutralizing effector functions are not completely distinct. In the RV144 adult HIV vaccine trial, non-neutralizing IgG antibody-dependent cellular cytotoxicity (ADCC)-associated antibodies were correlated with reduced risk of HIV infection [23]. Additionally, non-neutralizing antibody responses were correlated with protection induced by HIV vaccine candidates in adult RM models [51,52]. In infants, the presence of non-neutralizing antibodies capable of ADCC functions has been associated with better clinical outcomes, during breastfeeding [53]. Therefore, it might be postulated that upon HIV immunization at birth, prior to the development of bNabs, non-neutralizing antibody responses such as ADCC might confer some level of protection against breastmilk transmission (Fig 1). Interestingly, while only a short-lived ADCC response was detectable in infant HIV vaccinees from the PACTG 230 trial [24] (Table 1), durable HIV-specific ADCC responses were obtained in infant RMs immunized with an HIV vaccine [54], suggesting that further studies involving non-neutralizing antibody responses in the context of pediatric HIV vaccination are necessary.

## Modulating the early life immune landscape to augment protective anti-HIV immunity

Newborns transition from a relatively sheltered intrauterine environment to an environment with multiple antigenic exposures. To obtain survival benefits during the period of immune maturation, newborns establish a highly tolerogenic environment and exhibit a distinct immune profile than adults [reviewed in [16]]. Therefore, to optimally design a pediatric vaccine regimen tailored to the developing infant’s immune landscape, understanding earlylife immune ontogeny remains crucial.

### Use of age-relevant adjuvants

While alum has been the standard adjuvant of choice for commercial pediatric vaccines, HlV pediatric vaccine trials have reported the superiority of MF-59 adjuvant in mounting potent and durable antibody responses when compared to alum. In PACTG 230 trial, the MF-59-adjuvanted vaccine formulation was associated with durable anti-Env IgG responses, which was associated with higher breadth and durability as compared to the alum-adjuvanted counterpart [24]. This indicates that proper selection of adjuvants will be essential to augment infant vaccination responses. The ability of adjuvants to differentially modulate immune responses in different age groups has only recently started to be appreciated [55]. Since infants exhibit an intrinsic bias toward Th2 responses, an effective HIV vaccine might require adjuvants that enhance Th1 responses. Indeed, incorporation of the TLR7/8 agonist adjuvant 3M-052, which can prime antigen presenting cells (APCs) by producing interleukin 12 (IL-12), in alum-adjuvanted pneumococcal vaccines (PCV), facilitated Th1 differentiation and significantly enhanced antibody responses in infant RMs immunized at birth [56]. Similarly, a recent study in infant RMs demonstrated a potential advantage of TLR-based adjuvants, AS01-TLR4 agonist and 3M-052-TLR7/8 agonist, on the induction of robust HIV-specific antibody responses compared to squalene or alum [57], although the protective efficacy of those elicited HIV antibodies was not investigated. Therefore, further exploration of age-specific mechanisms of adjuvant effects will be required to develop potent and durable pediatric HIV vaccines.

### Alterations of the infant microbiome to optimize vaccine responses

Emerging evidence suggests that an individual’s microbiome can influence immune responses to vaccination [58]. Therefore, variations in microbial communities due to environmental, socioeconomic, and nutritional conditions partially explain the heterogeneity of an individual’s vaccine response [59]. While the mechanisms through which the microbiome modulate immune responses are likely complex and are not clearly defined, microbiota have been associated with a constant source of natural adjuvants that can shape one’s innate and adaptive immunity [60]. This endogenous adjuvant potential of microbiota was highlighted in a study where germ-free or antibiotic-treated mice had significantly impaired response to an inactivated influenza vaccine [61]. Additionally, an oral probiotic regimen augmented antibody response to multiple vaccines [62,63]. In contrast, the microbiome can also adversely impact vaccine efficacy by skewing antibody response toward non-protective antigens that resemble commensal bacterial antigens [reviewed in [64]]. In the setting of HIV vaccination, the preexisting B cell repertoire that develops against the commensal microbiota may divert the vaccine-elicited immune responses toward the gp41 region of vaccine candidates, as opposed to the more desirable neutralizing epitopes contained within the gp120 subunit [65]. Therefore, altering the existing gut microbiota in early infancy, when the B cell repertoire is predominantly naïve, could be beneficial to direct the immune response, upon vaccination, toward protective immunity. In fact, the first 2 years of life represents the perfect “window of opportunity” to perform microbial modulations, since the microbiome remains highly plastic during the time when maternal stimuli, nutrition, and introduction to solid foods, metabolism, and the environment are major contributors that shape microbial diversity [reviewed in [66]].

## Next-generation immunogens for pediatric vaccine trials

Neonates and infants possess unique immunological characteristics that promote the development of protective immunity via immunological and molecular pathways distinct from those of adults. Therefore, a deeper understanding of the infant immune system is needed to develop novel HIV immunization regimens tailored to the infant’s immune landscape. The fact that the most recent adult HIV vaccine trial (HVTN 702), done in South Africa, which tested a canarypox vector-based vaccine (ALVAC-HIV) with HIV subtype C gp120 protein adjuvanted with MF-59, was recently discontinued due to lack of efficacy [67] highlights the need to evaluate promising next-generation immunogens. To mimic the natural development of specific bNab lineages, a sequential immunization approach using HIV envelope sequences from patients who developed bNabs is currently being pursued in preclinical models [68,69]. Since in most cases natural evolution of a B cell lineage is unknown, an alternative approach is to design envelope constructs with specific antigenic features to target the bNab germline [70]. Epitope-based vaccine approaches consisting of envelope constructs that incorporate a portion of the bNab epitope to obtain a focused immune response are also currently being evaluated [reviewed in [71]]. The neonatal B cell compartment primarily consists of naïve and B cells [72] with high germinal center B cell activity, lower frequency of regulatory B cells, and limited diversity of B cell repertoire. Hence, early life may offer a unique opportunity to enhance B cell priming following vaccination, thereby providing potential advantages toward the development of bNabs. Additionally, infants demonstrate distinct B cell tolerance mechanism compared to adults [73,74], which could be a beneficial strategy of engaging and positively selecting B cells expressing specific germline immunoglobulin gene sequences. Other novel immunogen approaches that are currently being evaluated in adults include native-like envelope trimer immunogens [75], fold-on trimers [76], native flexible-linked (NFL) trimers [77], DNA vaccines encoding polyvalent gp120s [78], and mRNA vaccine [79]. While recent studies in adults have demonstrated the existence of bNab precursors [80], their frequency in pediatric population is largely unknown. Therefore, to assess whether pediatric immune system presents advantages for induction of neutralization breadth, novel immunization strategies need to be evaluated in pediatric preclinical models [81].

The pediatric HIV vaccine protocol HVTN 135 is currently in development to assess the safety and immunogenicity of HIV CH505 transmitted-founder (T/F) gp120 adjuvanted with the TLR4 agonist GLA-SE in HIV-exposed infants. This Phase I trial will use the CH505 T/F protein that is currently being tested in an adult HIV vaccine trial, and the results from HVTN135 will determine if this vaccine is safe to be used in infants. Additionally, this trial will indicate whether infants develop a distinct immune response to this vaccine as compared to adults, hence providing valuable information for the design of future pediatric HIV vaccine trials. Ultimately, additional clinical trials will be required to assess if immunization at birth can protect infants from vertical HIV transmission during infancy and against sexual HIV transmission during adolescence.
